# Temperature Sensing in Seawater Based on Microfiber Knot Resonator

**DOI:** 10.3390/s141018515

**Published:** 2014-10-08

**Authors:** Hongjuan Yang, Shanshan Wang, Xin Wang, Yipeng Liao, Jing Wang

**Affiliations:** Department of Physics, College of Information Science and Engineering, Ocean University of China, Qingdao 266100, China; E-Mails: yanghongjuan1989@gmail.com (H.Y.); wangshanshan@ouc.edu.cn (S.W.); wxinflying@foxmail.com (X.W.); oucliaoyipeng@hotmail.com (Y.L.)

**Keywords:** microfiber, knot resonator, seawater temperature, sensing, ocean internal-wave

## Abstract

Ocean internal-wave phenomena occur with the variation in seawater vertical temperature, and most internal-wave detections are dependent on the measurement of seawater vertical temperature. A seawater temperature sensor based on a microfiber knot resonator (MKR) is designed theoretically and demonstrated experimentally in this paper. Especially, the dependences of sensing sensitivity on fiber diameter and probing wavelength are studied. Calculated results show that sensing sensitivity increases with the increasing microfiber diameter with the range of 2.30–3.91 μm and increases with the increasing probing wavelength, which reach good agreement with results obtained by experiments. By choosing the appropriate parameters, the maximum sensitivity measured can reach to be 22.81 pm/°C. The seawater temperature sensor demonstrated here shows advantages of small size, high sensitivity, easy fabrication, and easy integration with fiber systems, which may offer a new optical method to detect temperature of seawater or ocean internal-wave phenomenon and offer valuable reference for assembling micro sensors used for other parameters related to seawater, such as salinity, refractive index, concentration of NO_3_^−^ and so on.

## 1. Introduction

Ocean internal-wave is an important ocean phenomenon [1]. Due to its randomness and damage to the safety of navigation, it is essential to monitor it real-time in oceanographic engineering and the marine military. Currently, the most widely used device for detecting ocean internal-wave is the conductivity-temperature-depth (CTD) system by measuring the seawater vertical temperature [2], which has the advantages of high precision and wide-ranging practicality. However, it cannot be used in detecting the seawater skin temperature and temperature of micro/nano scale due to its large size. Moreover, its expensive price and slow response greatly limits its application in ocean detection. Thus, it is urgent to develop a micro-structure seawater temperature sensor to meet the demands for fine-structure and other micro/nano scale detection in seawater.

In recent years, microfiber has attracted more and more attentions in optical sensing due to its compact size, low loss and high sensitivity [3–14]. Additionally, several microfiber temperature sensors have been fabricated successfully, including sensors based on microfiber knot resonator (MKR) [15–17], microfiber coil resonator (MCR) [18], taper microfiber [19–21], fiber bragg grating [22–24], fiber coupler [25,26] and Fabry–Perot interferometer [27]. These sensors can detect the temperature of air or heating platform, which show advantages of high sensitivity and fast response; however, to the best of our knowledge, the temperature sensor for liquid especially in seawater has not been demonstrated experimentally.

In this paper, a seawater temperature sensor based on a microfiber knot resonator (MKR) is proposed. The dependences of sensing sensitivity on fiber diameter and probing wavelength are studied theoretically, which show good agreement with experimental results. By choosing the appropriate parameters for assembling sensor, the maximum sensitivity measured can reach 22.81 pm/°C. The seawater temperature sensor demonstrated here shows the advantages of small size, high sensitivity, easy fabrication, and easy integration with fiber systems, and may offer a new optical method to detect temperature of seawater or ocean internal-wave phenomenon. Otherwise, other parameters related to seawater also can be detected using micro sensor based on MKR.

## 2. Theory

The MKR can be used for seawater temperature sensing due to the thermo-optical effect and thermal-expanding effect. Compared to the thermo-optical effect, thermal-expanding effect can be ignored [28]. The thermo-optical coefficients (TOCs) of the silica and seawater are about 10^−5^ [29] and −10^−4^ [30], respectively. With the variation of the seawater temperature, the refractive indices (RIs) of the silica and seawater change, and the effective refractive index *n_eff_* varies immediately, which results in the shift of the resonant wavelength. Thus, the sensitivity *S* of the sensor can be defined as the ratio of the shift of the resonant wavelength *λ* to the variation of the temperature *T*, which can be written as:

(1) $$S=\frac{\partial \lambda }{\partial T}=\frac{\partial \lambda }{\partial n_{\text{eff}}}\cdot \frac{\partial n_{\text{eff}}}{\partial T}=\frac{\lambda }{n_{\text{eff}}}\cdot \frac{\partial n_{\text{eff}}}{\partial T}$$

**Figure 1.**
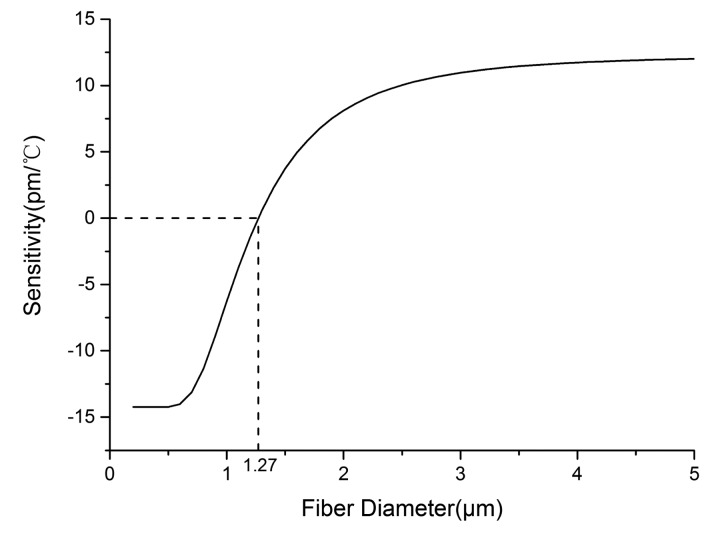
The simulated sensitivity dependence on fiber diameter.

**Figure 2.**
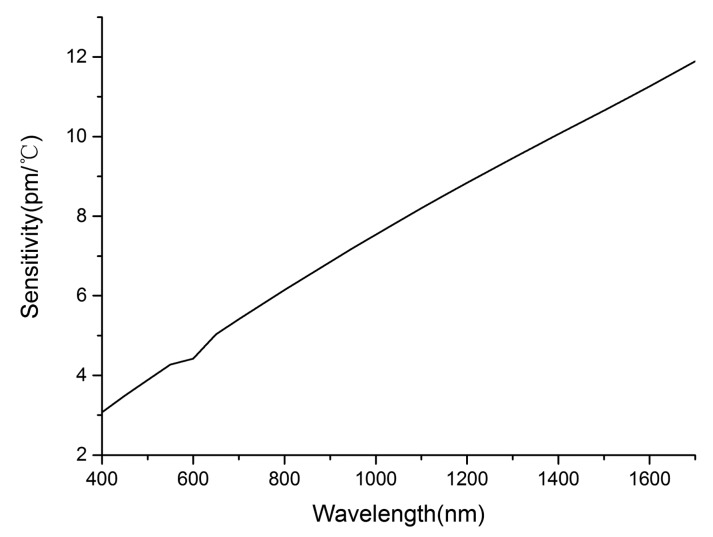
The simulated sensitivity dependence on probing wavelength.

Due to the fact that 97% of seawater is pure water and there is no research results of the relationship between the RI and temperature of seawater in infrared band, we use the RI of pure water [31] instead of the RI of seawater approximatively. The losses in the MKR, including guiding loss, bending loss, and absorption loss have no influence on the sensitivity of the sensor. Thus, the losses are not considered in theoretical calculation. Combining Formula (1) and the analytic equation of propagation constant [32], the dependences of sensitivity on fiber diameter and probing wavelength are obtained, as shown in Figures 1 and 2. The MKR diameter does not affect the sensitivity of the sensor, because sensitivity mainly relies on the strong evanescent field outside the microfiber [10].

As shown in Figure 1, with the fiber diameter of 0.20–1.27 μm, the sensitivity decreases with the increasing fiber diameter; however, with the fiber diameter of 1.27–4 μm, the sensitivity increases with the increasing fiber diameter; and with the fiber diameter larger than 4 μm, the sensitivity increases with the increasing fiber diameter slowly. In other words, the sensitivity changes little by increasing the fiber diameter when the fiber diameter is larger than 4 μm. The reason is that the silica and seawater show the opposite thermal response due to their opposite TOCs. The resonant wavelength shifts toward the long wavelength (red shift) when the positive TOC of silica dominates, and the resonant wavelength shifts toward the short wavelength (blue shift) when the negative TOC of seawater dominates. The microfiber with smaller diameter has more power transmitted in seawater due to the stronger evanescent field effect. The evanescent field and the influence of the negative TOC of seawater becomes weaker with increasing fiber diameter, which leads to the transformation of the resonant wavelength from red shift to blue shift. To obtain high sensitivity, microfibers with diameters ranging from 2.5 μm to 4 μm are chosen.

The dependence of sensitivity on probing wavelength is also studied with the 3-μm-diameter microfiber. As is shown in Figure 2, sensitivity increases linearly with increasing probing wavelength. It can be said that the TOC of seawater increases with increasing probing wavelength, which results in increasing sensitivity of the sensor.

Based on the above calculations, we can conclude that for a system used for seawater temperature sensing, a larger fiber diameter and longer probing wavelength are preferred. Next, experiments are carried out to experimentally demonstrate the temperature sensing and validate the theoretical results, simultaneously.

## 3. Experiment and Discussion

### 3.1. Experiment System

**Figure 3.**
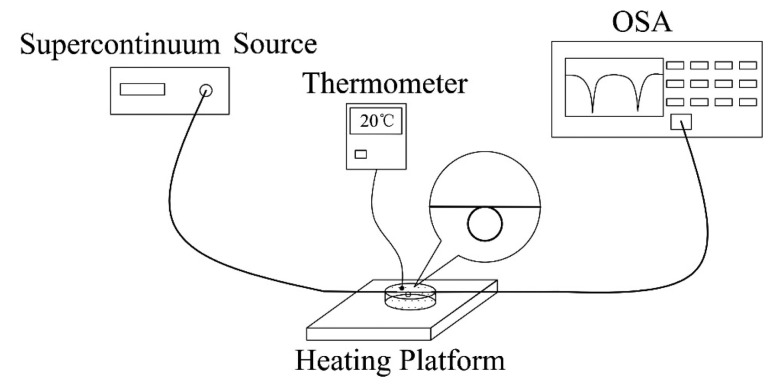
The schematic diagram of the experiment system.

The schematic diagram of the experiment system is shown in Figure 3. The instruments in the system include supercontinuum source (SuperK™ Compact), optical spectrum analyzer (AQ6370C), temperature-controlled heating platform and thermocouple thermometer. The MKR is prepared under an optical microscope with the aid of fiber probe as follows: firstly, microfiber is fabricated by flame stretching method from standard single mode fiber. Then, the freestanding end of the fiber is fabricated into a relatively large loop about a few millimeters in diameter, which is then tightened into a smaller knot by pulling the free end of the fiber. After this manipulation, a MKR is obtained [33]. One side of the MKR is connected with the supercontinuum source, and the other side is coupled to the optical spectrum analyzer for signal collection. Finally, the assembled MKR is immersed in seawater and the vessel of seawater is placed on the heating platform. The seawater temperature is varied by changing the temperature of heating platform, and the real time temperature is monitored by the thermocouple thermometer. The salinity of seawater sample used in the experiment is 34‰, which was measured by salinity meter (PR-100SA).

### 3.2. Seawater Temperature Experiment

**Figure 4.**
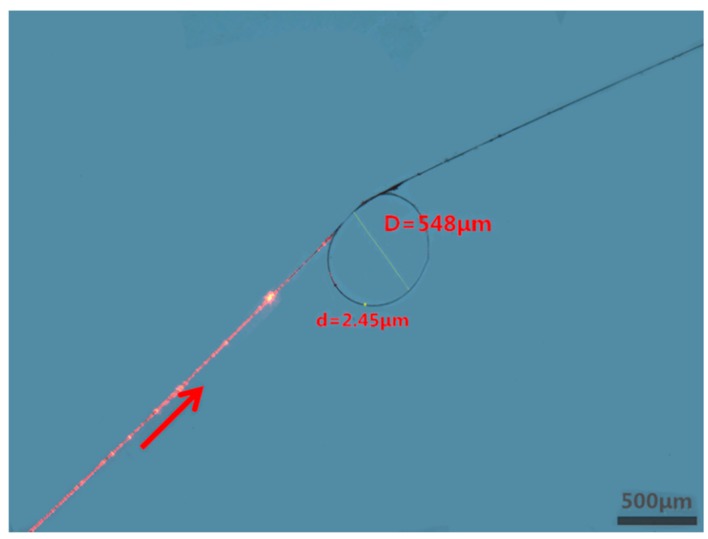
The optical microscope image of a 548-μm-diameter MKR with a 2.45-μm-diameter microfiber.

**Figure 5.**
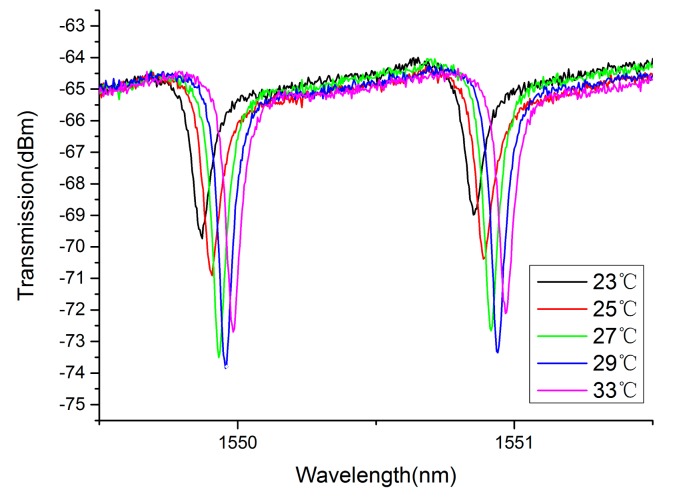
The resonant spectra at different temperatures with 2.45-μm-diameter fiber.

**Figure 6.**
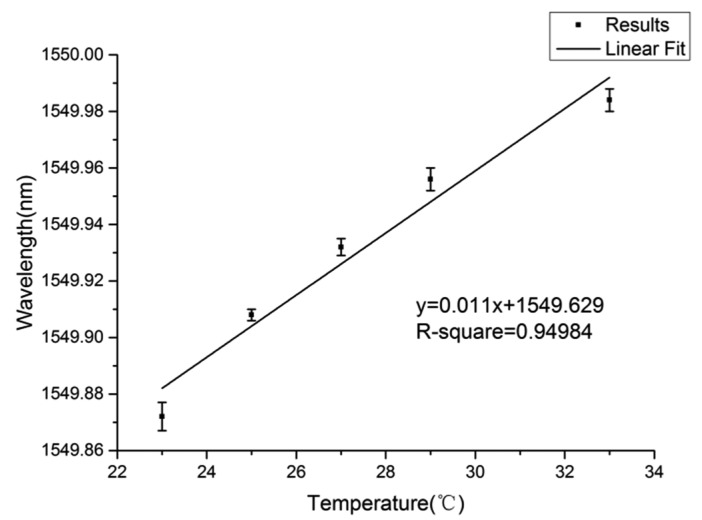
The relationship between resonant wavelength and temperature and the linear fitting with 2.45-μm-diameter fiber. The error bars show the differences between experimental and fitting results.

To demonstrate the temperature sensing in seawater, a 548-μm-diameter knot resonator is assembled using a 2.45-μm-diameter silica microfiber, an optical microscope image of which is shown in Figure 4. The symbol arrow shows the direction of red light, which acts as an indication light. The insertion loss of the MKR is 17.6 dB, which is mainly attributed to the bending loss and absorption loss [34]. For optical characterization, a circularly polarized broadband light from a supercontinuum source is launched. The initial temperature is recorded to be 23 °C and the temperature is increased gradually by the heater. Figure 5 shows the transmission spectrum of this resonator with some typical temperatures. As is shown, the resonant wavelength shifts toward the long wavelength with the increasing temperature. Compared to the negative TOC of seawater, the positive TOC of silica is dominant in the 2.45-μm-diameter fiber. The peak values of resonant wavelength at different temperatures are plotted in Figure 6. It can be seen that the wavelength shift increases approximately linearly with the increase of temperature with a slope of about 11.00 pm/°C, which indicates that the measured sensitivity of this sensor is about 11.00 pm/°C.

It is noticed that besides the resonance wavelength shift, the line shape of the spectra changes with the temperature, as shown in Figure 5. It can be said that the changes of RIs result in the variation in loss at different temperatures, which affects the coupling between MKR and the single mode fiber [35]. Thus, the line width and depth vary with increasing temperature. Additionally, the changes of RIs also affect the resonance properties and output intensity, which is another reason for the changes of line shape.

To further investigate the dependence of sensing sensitivity on different fiber diameters, six MKRs were fabricated. The fiber diameters and ring diameters are measured by microscope and their *Q*-factors and finenesses under the same wavelength of 1550 nm are listed in Table 1.

**Figure 7.**
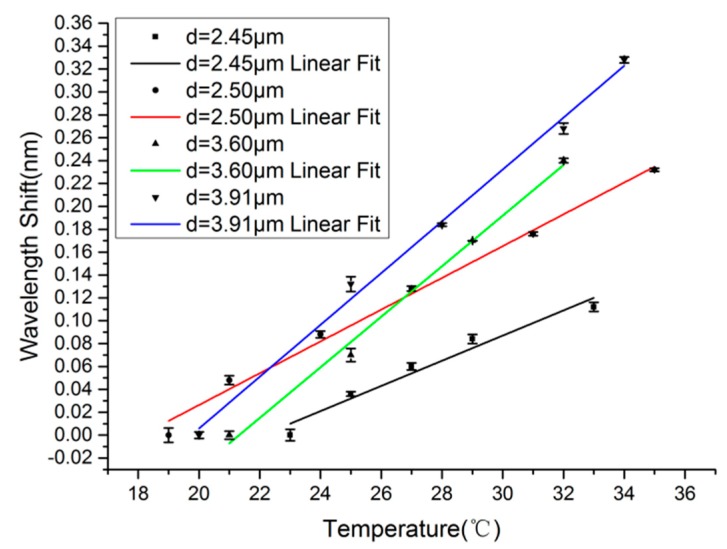
The linear fittings of resonant wavelength with temperature for MKRs with fiber diameters of 2.45-, 2.50-, 3.60- and 3.91-μm. The error bars show the differences between experimental and fitting results.

**Figure 8.**
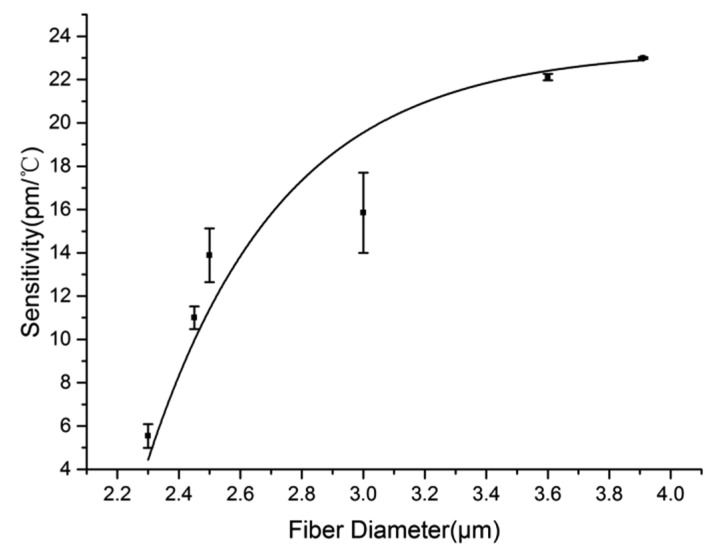
The dependence of sensitivity on fiber diameter. The error bars show the differences between experimental and fitting results.

By a similar measuring method to that used in Figure 5, the linear fittings of resonant wavelength with temperature for MKRs with fiber diameters of 2.45-, 2.50-, 3.60- and 3.91-μm are shown in Figure 7. The measured sensitivities of these MKRs are 11.00, 13.89, 22.11 and 22.65 pm/°C, respectively, which indicates that larger fiber diameter results in higher sensitivity. To further show the dependence of sensitivity on fiber diameter, we plot their relationship in the fiber diameter range of 2.30–3.91 μm, as is shown in Figure 8. It can be seen clearly that the sensitivity of the sensor increases with the increasing fiber diameter, which is consistent with the calculated results.

**Figure 9.**
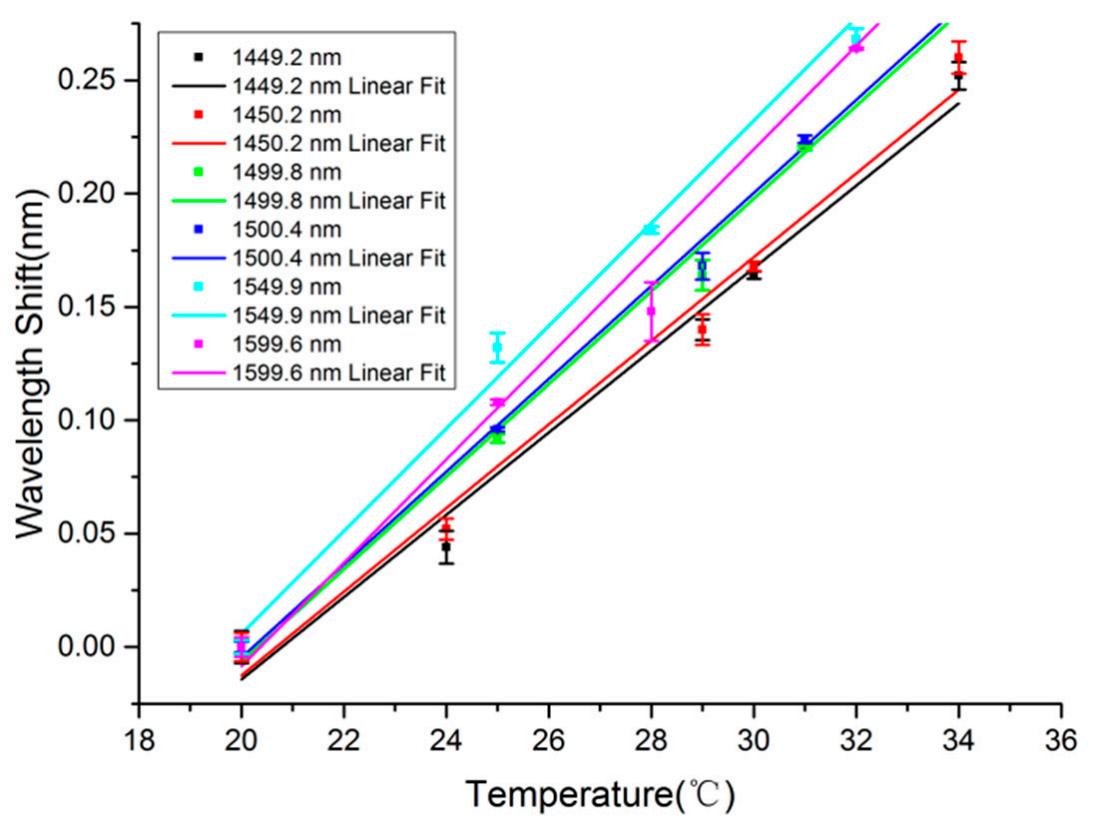
The linear fittings of resonant wavelength with temperature for MKR with 3.91-μm-diameter microfiber under probing wavelengths of 1449.2, 1450.2, 1499.8, 1500.1, 1549.9 and 1599.6 nm. The error bars show the differences between experimental and fitting results.

**Figure 10.**
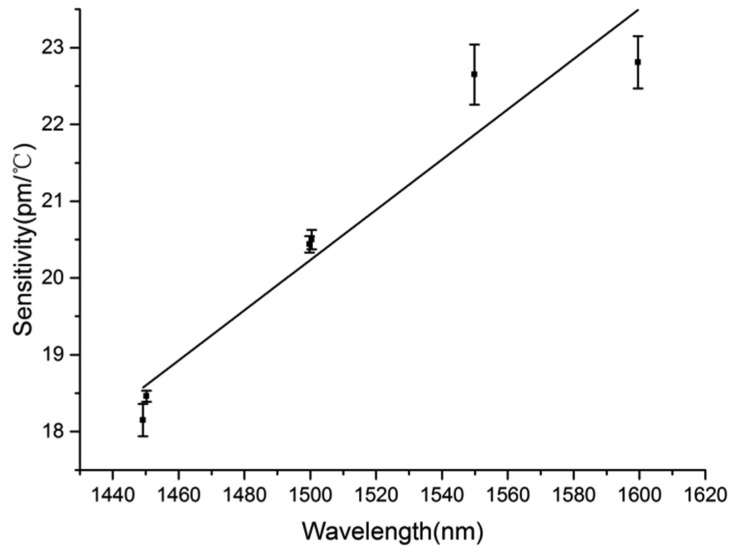
The dependence of sensitivity on probing wavelength. The error bars show the differences between experimental and fitting results.

Similar to the fiber diameter, probing wavelength is an equivalent parameter that determines the sensitivity. To reveal the dependence of sensitivity on probing wavelength, we firstly measure the wavelength shift with the temperature to obtain the sensing sensitivity of a 473-μm-diameter MKR with 3.91-μm-diameter microfiber around probing wavelengths of 1449.2, 1450.2, 1499.8, 1500.1, 1549.9 and 1599.6 nm, respectively. Correspondingly, the measured sensitivities are estimated by the slopes of the fitting lines shown in Figure 9, which are 18.15, 18.46, 20.44, 20.5, 22.65 and 22.81 pm/°C, respectively. Based on the sensitivities, we plot the dependence of sensitivity on the probing wavelength in Figure 10. It is obvious that sensitivity increases approximately linearly with the increase of wavelength, which is also consistent with the simulated results shown in Figure 2.

## 4. Conclusions

In conclusion, a seawater temperature sensor based on a microfiber knot resonator (MKR) is designed theoretically and demonstrated experimentally in this paper. The dependences of sensitivity on microfiber diameter and probing wavelength are studied. Results show that sensing sensitivity increases with the increasing microfiber diameter with the range of 2.30–3.91 μm, which is due to the fact that the microfiber with a smaller diameter transmits more power in seawater because of a stronger evanescent field effect. The evanescent field and the influence of the negative TOC of seawater weaken with increasing fiber diameter, and results in increasing sensitivity. In addition, sensitivity also increases with increasing probing wavelength because of increases in the negative TOC of seawater. With a fiber diameter of 2.3–3.91 μm, and a probing wavelength of 1550–1600 nm, the measured sensitivity reaches levels of 5.540–22.81 pm/°C. The theoretical and experimental results agree well. Results obtained in this paper offer helpful reference for choosing the appropriate parameters for assembling seawater temperature sensor. By choosing the appropriate parameters for assembling sensor, the maximum sensitivity measured can reach 22.81 pm/°C. The seawater temperature sensor demonstrated here show the advantages of small size, easy fabrication, and easy integration with fiber systems, and may offer a new optical method to detect the temperature of seawater or ocean internal-wave phenomenon and offer valuable reference for assembling micro sensors used for other parameters related to seawater, such as salinity, concentration of NO_3_^−^ and so on.

## Figures and Tables

**Table 1.** The Q factor and fineness of MKRs under the same wavelength of 1550 nm.

**NO.**	**Fiber Diameter d (μm)**	**MKR Diameter D (μm)**	**Q-Factor**	**Fineness**
	2.45	548	2.6 × 10^4^	16.4
	2.3	1161	4.8 × 10^4^	14.5
	2.5	492	1.8 × 10^4^	12.45
	3.0	1104	2.3 × 10^4^	7.39
	3.6	173	0.5 × 10^4^	10.76
	3.91	473	3.0 × 10^4^	11.69
